# Health-related quality of life and unmet healthcare needs in Huntington’s disease

**DOI:** 10.1186/s12955-016-0575-7

**Published:** 2017-01-07

**Authors:** Marleen R. van Walsem, Emilie I. Howe, Gunvor A. Ruud, Jan C. Frich, Nada Andelic

**Affiliations:** 1 Centre for Habilitation and Rehabilitation Models and Services (CHARM), Institute for Health and Society, University of Oslo, P.O. Box 1130, Blindern, 0318 Oslo Norway; 2 Department of Neurohabilitation, Oslo University Hospital, P.O. Box 4950, Nydalen, 0424 Oslo Norway; 3 Department of Physical Medicine and Rehabilitation, Oslo University Hospital, P.O. Box 4950, Nydalen, 0424 Oslo Norway; 4 Centre for Rare Disorders, Oslo University Hospital, Rikshospitalet, P.O. Box 4950, Nydalen, 0424 Oslo Norway; 5 Institute of Health and Society, University of Oslo, P.O. Box 1130, Blindern, 0318 Oslo Norway; 6 Department of Neurology, Oslo University Hospital, P.O. Box 4950, Nydalen, 0424 Oslo Norway

**Keywords:** Huntington’s disease, Health-related Quality of Life, Healthcare needs, Healthcare services, EQ-5D, NPCS

## Abstract

**Background:**

Huntington’s disease (HD) is a rare neurodegenerative disorder with a prevalence of 6 per 100.000. Despite increasing research activity on HD, evidence on healthcare utilization, patients’ needs for healthcare services and Health-Related Quality of Life (HRQoL) is still sparse. The present study describes HRQoL in a Norwegian cohort of HD patients, and assesses associations between unmet healthcare and social support service needs and HRQoL.

**Methods:**

In this cross-sectional population-based study, 84 patients with a clinical diagnosis of HD living in the South-East of Norway completed the HRQoL questionnaire EuroQol, EQ-5D-3L. Unmet needs for healthcare and social support services were assessed by the Needs and Provision Complexity Scale (NPCS). Furthermore, functional ability was determined using the Unified Huntington’s Disease Rating Scale (UHDRS) Functional assessment scales. Socio-demographics (age, gender, marital status, occupation, residence, housing situation) and clinical characteristics (disease duration, total functional capacity, comorbidity) were also recorded. Descriptive statistics were used to describe the patients’ HRQoL. Regression analyses were conducted in order to investigate the relationship between unmet healthcare needs and self-reported HRQoL.

**Results:**

The patients were divided across five disease stages as follows: Stage I: *n* = 12 (14%), Stage II: *n* = 22 (27%), Stage III: *n* = 19 (23%), Stage IV: *n* = 14 (16%), and Stage V: *n* = 17 (20%). Overall HRQoL was lowest in patients with advanced disease (Stages IV and V), while patients in the middle phase (Stage III) showed the most varied health profile for the five EQ-5D-3L dimensions. The regression model including level of unmet needs, clinical characteristics and demographics (age and education) accounted for 42% of variance in HRQoL. A higher level of unmet needs was associated with lower HRQoL (β value - 0.228; *p* = 0.018) whereas a better total functional capacity corresponded to higher HRQoL (β value 0.564; *p* < 0.001).

**Conclusions:**

The study findings suggest that patients with HD do not receive healthcare services that could have a positive impact on their HRQoL.

## Background

Huntington’s disease (HD) is a rare neurodegenerative disorder with a prevalence of 6 per 100.000 in European, North American and Australian populations [1]. This chronic and complex disease is characterized by a triad of symptoms, including motor impairment, decline in cognitive function, and psychiatric disturbances. Symptoms develop gradually and result in progressive functional decline, and a complex and continuously changing clinical picture [2]. Although a clinical diagnosis of HD is based on the presence of undisputable motor symptoms, psychiatric symptoms and changes in cognitive function may precede clinical diagnosis by several years [3–6]. Most patients receive clinical diagnosis in mid adult life (between 30 and 50 years of age), with an estimated disease duration of 17 – 20 years [7].

Clinical care of patients with HD is focused on disease management, alleviating symptoms and maintaining functional ability and health-related quality of life (HRQoL) [7]. Treatment requires multidisciplinary, comprehensive care from several groups of healthcare professionals, and may include both pharmacological and non-pharmacological interventions, as curative treatment currently does not exist [8–11]. HRQoL has emerged as an increasingly important patient and clinician reported outcome measure alongside other endpoints of symptom ratings of HD [12, 13]. Furthermore, the establishment of several large observational studies during the last 5 to 10 years, such as REGISTRY and PREDICT-HD [14, 15] has led to rapidly increasing research activity on HRQoL in HD. HRQoL is a multidimensional concept reflecting impact of disease and/or its treatment on an individuals’ physical, emotional and social well-being [16]. Research has found lower HRQoL in patients with clinical HD compared to HD premotor manifest patients, persons at risk for HD and their partners [17]. Furthermore, studies have aimed at identifying which disease related factors most strongly correlate with HRQoL in patients with HD [10, 11, 17, 18]. Two studies found that functional ability and depression were strongly associated with a decline in HRQoL in HD patients [11, 17, 18]. Read et al. [17] found that neuropsychiatric symptoms and cognitive impairment had the strongest negative relationship with HRQoL. One study identified depression and cognitive impairment as the strongest determinants of HRQoL [10]. A reason for the slight differences in findings may be due to the studies comprising of different populations of HD (i.e. only HD patients with early HD).

In recent years, there has been an increased focus on the association between unmet health care needs and HRQoL in health services research [19]. Despite general knowledge of the need for multidisciplinary and comprehensive healthcare for patients with HD, there is a lack of research investigating the relationship between healthcare service delivery (or lack thereof) and HRQoL. One previous study has shown that HD patients have a considerable level of unmet needs for healthcare and social support, indicating that many patients do not receive the comprehensive care they need [20]. To the authors’ knowledge, only one study that included patients with rare long-term neurological conditions, including HD, has investigated both HRQoL *and* healthcare services, more specifically access to supportive health and social care. In addition to providing support to previous studies, indicating reduced HRQoL in HD patients compared to the general population and other diseases, the study suggested that patients with rare complex neurological disorders do not use health and social care services that could have a positive effect on their HRQoL [21]. No studies specifically investigating the potential association between gaps in healthcare service needs and provision and HRQoL in HD have been performed.

Thus, the aims of this study are:

- To describe the health status (HRQoL) in a Norwegian cohort of patients with HD.
- To assess the association between unmet needs for healthcare and social support services and HRQoL.

We expected to find a higher level of unmet needs for health care services to be associated with lower HRQoL.

## Methods

### Participants and participant recruitment

Patients with a clinical diagnosis of HD living in the South-Eastern region of Norway (population 2.7 million inhabitants) were invited to participate in a survey. Eligible patients were identified and recruited through the regional academic medical center, Oslo University Hospital, through the Department of Neurology, Department of Neurohabilitation, Department of Medical Genetics and through the national advisory service for HD, the Centre for Rare Disorders. Additionally, Vikersund Rehabilitation Centre, offering a rehabilitation program for patients with HD, provided invitations to additional patients. Furthermore, we collaborated with a Norwegian professional network for community care in HD (Fagnettverk Huntington) and the Norwegian HD lay association (Landsforeningen for Huntingtons sykdom), in order to attempt reaching as many eligible patients as possible. We identified 158 eligible patients (corresponding to a prevalence of 5.9/100.000). A written invitation enclosing study information and an informed consent form was sent to these patients. Informed consent was obtained from 88 of the invited patients. Two patients were excluded after careful review of the patients’ medical records by a medical expert (JF), as we questioned if the patients had sufficient clinical symptoms to formally have a clinical diagnosis at the time of inclusion.

### Ethics

The study was approved by the Regional Ethical Committee (ref. 2013/2089). Informed consent was obtained for all patients prior to inclusion in the study. Consent was obtained from the primary caregiver or legal representative for patients who were unable to give informed consent themselves.

### Data collection

Data were collected during study visits either as outpatient study visits (39%) or in patients’ homes (61%). Appointments for study visits were made by contacting the patient/carer upon receiving the informed consent form. Socio-demographic, clinical and disease specific data were collected and a clinical rating and needs assessment was performed as part of a survey interview with the patient and/or primary carer. Data collection and assessments were conducted by the same two experienced clinical raters (MRvW & EIH). Additional information from the patients’ medical records was used to estimate years of education and level of education (lower vs. higher) for four patients and occupational type (manual vs. non-manual) for three patients. We estimated disease duration (number of years with clinical diagnosis of HD) using clinical information that was available through the patients’ medical records for three patients, as we were unable to collect this information at the study visit. We were unable to obtain information concerning the number of CAG repeats in the HTT-gene for three patients. Next, patients were rated regarding their functional ability and their needs for healthcare and social services. At the end of the visit patients were asked to report their HRQoL by completing a generic questionnaire for HRQoL. If the patients were unable to independently fill in the questionnaire (i.e. due to motor impairment), their primary carer assisted them. They were explicitly informed that the questionnaire was a self-report measure aiming to reflect the patients rating of their health status. The carer assisted the patient in indicating their choice on the form in case of motor impairment, or aided the patient by reading or explaining the questions. For eight patients with advanced disease their primary carer completed the questionnaire on behalf of the patient. These primary carers were instructed to reflect the patients’ experienced health status and HRQoL as well as possible. If they were unable to do so the questions were kept open and became missing values. All carers were either family members or health care personnel involved with patients on a daily basis. When questionnaires were not completed during the study visit, they were returned using a prepaid reply envelope. Patients, who had not returned the questionnaire by the end of the inclusion period, were followed up by telephone.

### Measurements

The patients’ functional ability was evaluated with the three scales of the Unified Huntington’s Disease Rating Scale (UHDRS) - Functional assessment including the: a) Total Functional Capacity Scale (TFC) with a scoring range of 0–13, b) the Functional Assessment scale (FAS), a checklist for daily living, range 0–25 and c) the Independence Scale (IS), indicating the level of independence in %, scoring range 0–100. The TFC is used to classify HD patients into five functional disease stages: Stage I corresponds to a TFC score of 11–13, Stage II to a TFC score of 7–10, Stage III to a TFC score of 3–6, Stage IV to a TFC score of 1–2 and Stage V to a TFC score of 0. Higher scores on these scales indicate higher functioning, corresponding to higher independence [22].

In order to rate the level of unmet needs for healthcare and social services, we used the Needs and Provision Complexity Scale (NPCS), clinician version [23]. This tool is recently developed in the UK in order to identify healthcare and social support needs among patients with long term neurological conditions. It measures the patients’ needs for healthcare and support services, Part A (NPCS-Needs) and to which extend these needs are met through service provision Part B (NPCS-Gets). In the clinician version, needs (Part A) are assessed in a systematic and normative way by the clinician, and part B is systematically recorded by the clinician based on information provided by the patient and/or carer. The measure includes 15 items with a total scoring range of 0–50 covering low and high levels of needs. The items are divided over 6 sub-scales representing two domains: a) Health and Personal care needs and b) Social and support needs, both having a score range of 0–25. The NPCS can be used at the population level to identify gaps in health service provision. At the individual level the NPCS can be used to monitor the changing needs and provisions of patients along the care pathway over time. The scale has shown good psychometric qualities and has been translated to Norwegian [20, 24].

We used the EQ-5D-3L questionnaire to measure HRQoL. This is a generic self-report measure developed by the EuroQol Group [25]. The measure is used in several health conditions, including HD [26–29]. The scale consists of two parts. The first part comprises five single item dimensions of health: mobility, self-care, usual activities, pain/discomfort and anxiety/depression, which can be rated on three levels of severity representing no problem (1), slight problem (2), major problem (3). The level scores for these five dimensions can be presented in health profiles as well as global health indices with a weighted total value for HRQoL. Part two is a Visual Analogue Scale (VAS) ranging from 0 (worst health-state) to 100 (best health state), and is often used as a general measure for HRQoL. The EQ-5D 3L has been found valid to use in the Norwegian population [30]. For the purpose of our study we use the level scores in order to describe health profiles for the five disease stages and the VAS scores as an overall measure of perceived HRQoL.

### Statistical analyses

Descriptive statistics of mean values and standard deviation (SD) (normally distributed variables) and median values with interquartile range (IQR) (non-normally distributed variables) were calculated for socio-demographic and clinical sample characteristics and the total level of unmet needs and VAS-score (HRQoL) for the complete sample and each of the five disease stages. Overall group differences between disease stages were computed using one-way ANOVA (normally distributed continuous variables) and Kruskal-Wallis for K-samples (non-normally distributed variables). Group differences for nominal variables were calculated using cross-tabulation Chi-square tests. NPCS total levels of unmet needs were calculated as the discrepancy between the total level of Needs and Gets: NPCS Needs score – NPCS Gets score = NPCS Unmet needs score. In order to describe the health-status for the complete sample and each of the five disease stages, n and % with level scores of 1, 2 and 3 for each of the five dimensions were calculated, resulting in a health-status profile.

Regression analyses were performed in order to investigate the relationship between the total level of unmet needs and the patients’ overall self-reported HRQoL. Data were inspected for violation of assumptions, which resulted in logarithmical transformation of the scores for total level of unmet needs and disease duration. The relationship between each of the independent variables (level of unmet needs and socio-demographic and clinical variables) and the dependent variable HRQoL was investigated using simple linear regression. The independent variable Informant was collapsed to a dichotomous variable grouping patient alone (*N* = 27) vs. patient with informant or informant only (*N* = 57). Four disease-related variables (disease duration, TFC, informant and housing situation) and the independent variable of interest, total level of unmet needs reached levels of significance. All variables, with exception of housing situation due to the high correlation with TFC score, were entered into the regression analyses as control variables. Additionally, despite lacking significance in simple regression, comorbidity and education as these were shown to be associated with levels of unmet needs [20], and age known to influence HRQoL in the normal population, were entered in regression analyses as control variables. Multiple regression analyses were performed using a hierarchical approach (a block-wise analysis). Independent variables were entered in three blocks divided according to the variable of interest, total level of unmet needs for healthcare and social support services (block 1), clinical and disease related variables, including disease duration, TFC score, informant, and comorbidity (block 2) and socio-demographic variables, age and education (block 3). Results are presented in Adjusted *R* ^*2*^ and *R* ^*2*^ Change, and standardized Beta (β) values with confidence intervals. The direction of Beta value was expected negative for total level of unmet needs indicating lower levels of unmet needs corresponding to higher HRQoL. Prior to carrying out the multiple regression analyses, possible multicollinearity between independent variables was investigated using variance inflation factor (VIF). We examined influential data points using Cook’s distance. Variables with correlation coefficients > .70 were not included in the analyses. Residual analyses were performed and no outliers on any of the variables included in the analyses were identified. Levels of significance were set at p = 0.05 and all statistical tests were two sided. Statistical analyses were performed using SPSS version 21.0; SPSS Inc. Chicago IL.

## Results

### Participants’ socio-demographic and clinical characteristics

84 out of the 86 participants (97.7%) (53.2% of the 158 eligible patients) included in the survey study filled out the EQ-5D-3L questionnaires, and were included in the data-analyses. The mean age was 56.7 (SD 11.4) years. The patients were divided across five disease stages as follows: Stage I: *n* = 12 (14%), Stage II: *n* = 22 (27%), Stage III: *n* = 19 (23%), Stage IV: *n* = 14 (16%), and Stage V: *n* = 17 (20%). Socio-demographic characteristics for the complete sample and for each disease stage are presented in Table 1.

**Table 1:** Socio-demographic statistics for total sample and divided across disease stages

Variables	Categories	Complete sample (*N* = 84)	Stage I (*n* = 12)	Stage II (*n* = 22)	Stage III (*n* = 19)	Stage IV (*n* = 14)	Stage V (*n* = 17)	*p*-value
		Mean (SD)	Mean (SD)	Mean (SD)	Mean (SD)	Mean (SD)	Mean (SD)	
Age^b^		56.7 (11.4)	49.8 (9.5)	54.6 (12.9)	58.9 (11.1)	61.1 (11.5)	57.8 (9.0)	0.084
Education (years)^b^		12.9 (3.5)	14.3 (3.3)	13.8 (3.8)	11.7 (3.2)	12.5 (3.7)	12.4 (3.3)	0.179
		*n* (%)	*n* (%)	*n* (%)	*n* (%)	*n* (%)	*n* (%)	*P*-value (2-sided)
Gender	Female	37 (44)	5 (42)	8 (36.4)	7 (37)	7 (50)	10 (59)	0.616
	Male	47 (56)	7 (58)	14 (63.6)	12 (63)	7 (50)	7 (41)	
Education	Lower (≤12 years)	51 (60.7)	5 (42)	11 (50)	15 (79)	9 (64.3)	11 (65)	0.221
	Higher (>12 years)	33 (39.3)	7 (58)	11 (50)	4 (21)	5 (45.7)	6 (35)	
Civil status	Single	36 (42.9)	4 (33)	7 (31.8)	9 (47)	8 (57.1)	8 (47)	0.560
	Married	48 (57.1)	8 (67)	15 (68.2)	10 (53)	6 (42.9)	9 (53)	
Occupation^a^	Manual	40 (47.6)	5 (42)	9 (40.1)	12 (63)	6 (46.1)	8 (47)	0.643
	Non-manual	41 (48.8)	7 (58)	13 (59.1)	7 (37)	7 (53.8)	7 (41)	
Occupational status	Employed	14 (16.7)	11 (92)	3 (13.6)	0 (0)	0 (0)	0 (0)	0.000
	Unemployed	70 (83.3)	1 (8)	19 (86.4)	19 (100)	14 (100)	17 (100)	
Housing situation	Living at home	52 (61.9)	12 (100)	22 (100)	13 (68)	5 (35.7)	0 (0)	0.000
	Not living at home	32 (38.1)	0 (0)	0 (0)	6 (32)	9 (64.3)	17 (100)	
Residence	Rural	12 (14.3)	1 (8)	3 (13.6)	2 (10.5)	3 (21.4)	3 (18)	0.859
	Urban	72 (85.7)	11 (92)	19 (86.4)	17 (89.5)	11 (78.6)	14 (82)	
Informant	Patient	27 (32.1)	9 (75)	14 (63.6)	4 (21)	0 (0)	0 (0)	0.000
	Patient & informant	49 (58.3)	3 (25)	8 (36.4)	15 (79)	14 (100)	17 (100)	
	Informant only	8 (9.6)	0 (0)	0 (0)	0 (0)	0 (0)	0 (0)	

*SD* Standard deviation; ^a^3 responses missing (1 in Stage IV and 2 in stage V); ^b^using ANOVA, all other variables Chi-square

Overall group differences across disease stages were significant for all clinical characteristics (*p* < 0.001) except for comorbid conditions (*p* = 0.143) (having no comorbid conditions vs. having comorbid conditions) (see Table 2). As expected, patients in advanced disease stages have longer disease duration compared to patients in the early disease stages, while FAS total scores and IS scores declined from disease stage I to V, with scores in stage I being close to normal (FAS median (IQR) = 24 (2) & IS mean (SD) = 95.8 (±5.1)) and very affected in stage V (FAS median (IQR) = 0 (2) & IS mean (SD) = 20.9 (±5.7)).

**Table 2:** Clinical characteristics and NPCS scores for the total sample and all disease stages

Variables		Complete sample (*N* =84)	Stage I (*n* = 12)	Stage II (*n* = 22)	Stage III (*n* = 19)	Stage IV (*n* = 14)	Stage V (*n* = 17)	Sign.
		Median (IQR)	Median (IQR)	Median (IQR)	Median (IQR)	Median (IQR)	Median (IQR)	
Disease duration		6 (7)	2 (2)	5 (6)	7 (5)	8 (7)	11 (7)	*P* < 0.001
Total FAS score		15 (17)	24 (2)	20 (2)	15 (4)	5 (3)	0 (2)	*P* < 0.001
Independence score^a^		60 (26.5)	95.8 (5.1)	79.1 (2.9)	64.7 (6.3)	40.4 (10.8)	20.9 (5.7)	*P* < 0.001
		*n* (%)	*n* (%)	*n* (%)	*n* (%)	*n* (%)	*n* (%)	*P*-value (2-sided)
Comorbid conditions	No (ne)	48 (57.1)	7 (58)	9 (41)	9 (47)	10 (71)	13 (76)	0.143
	Yes	36 (42.9)	5 (42)	13 (59)	10 (53)	4 (29)	4 (24)	
NPCS variables		Median (IQR)	Median (IQR)	Median (IQR)	Median (IQR)	Median (IQR)	Median (IQR)	Sign.
Total score^b^	Needs	21 (17)	7.5 (6)	11 (6)	23 (12)	29 (7)	28 (10)	*P* < 0.001
	Gets	13 (16)	3 (3)	6.5 (8)	13.5 (12)	21 (7)	21 (10)	*P* < 0.001
	Unmet needs	6 (6)	3 (4)	5 (4)	8 (7)	6.5 (10)	6 (9)	*P* = 0.013
Domain score Health and personal care	Needs	11 (7)	6 (5)	7.5 (5)	11.5 (5)	13.5 (5)	14 (3)	*P* < 0.001
	Gets	8 (6)	2.5 (2)	5 (6)	6.5 (6)	9 (4)	9 (7)	*P* < 0.001
	Unmet Needs	3 (4)	2 (3)	2.5 (3)	5 (4)	4 (5)	4 (6)	*P* = 0.028
Domain score Sosial care and support^c^	Needs	10 (10)	2 (3)	4 (2)	11 (7)	13.5 (8)	14 (2)	*P* < 0.001
	Gets	5 (10)	0 (1)	1.5 (2)	5.5 (6)	11.5 (4)	11 (3)	*P* < 0.001
	Unmet needs	2 (3)	1 (3)	2 (2)	2.5 (5)	2 (6)	2 (4)	*P* = 0.053

*FAS* Functional Assessment Scale, *IQR* Interquartile range, ^a^normally distributed: reported mean (sd) and Anova; ^b^one missing from stage III; ^c^one missing from stage III; Needs = NPCS Part A; Gets = NPCS Part B

A significant group difference was found for total level of unmet needs for healthcare and social support services (p = 0.013). The level of unmet needs peaked in stage III (median = 8 (IQR = 7)). The levels of unmet needs for patients in stages I and II were lowest (median values and IQR were 3 (4) and 5 (4) respectively), while levels of unmet needs in stage IV and V were constant and higher than stages I and II, but lower than stage III, with median values and IQR of 6.5 (10) and 6 (9), respectively. Median scores and interquartile ranges for Needs (Part A), Gets (Part B) and Unmet needs for total unmet needs, and for the two domain scores “health and personal care”, and “social care and support” are also reported in Table 2.

### HRQoL of the total sample and across disease stages

**Fig. 1:**
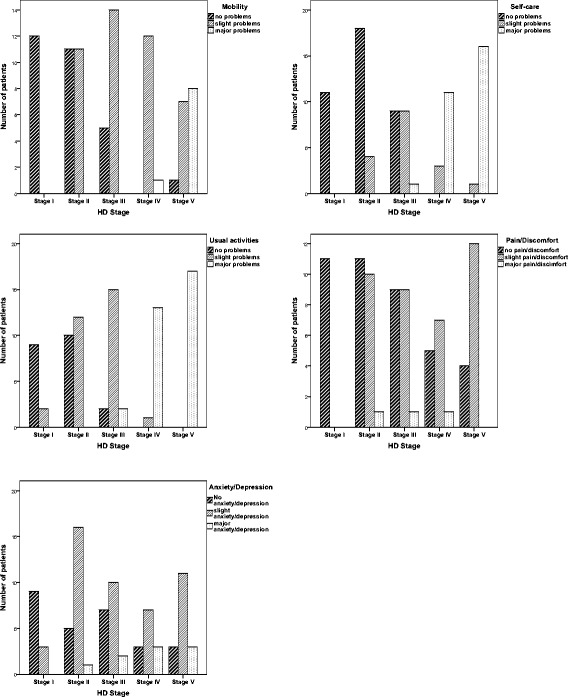
Bar-graphs showing health profiles of HD patients in Stage I to Stage V

Figure 1 shows bar graphs of the health profiles for the five disease stages, illustrated by the level scores for each of the five EQ-5D dimensions. In general, the health profile is most wide-ranging for patients in stage III, showing level scores across the whole range (1 (no problems) to 3 (major problems)) for each of the five dimensions except for *mobility*. Approximately two thirds of the total sample report slight or major problems (*n* = 53 (65.4%)) for *mobility*. Major problems are all reported in advanced disease (*n* = 1 (7%)) in Stage IV and *n* = 8 (47%) in Stage V). For the dimension *self-care*, more than half of all patients report slight or major problems (*n* = 45 (55.6%)). Major problems are mainly reported in advanced disease (Stage IV: *n* = 11 (78.6%), Stage V: *n* = 16 (94.1%)), except for 1 patient in stage III. Three quarters of the patients have slight or major problems for *usual activity* (*n* = 62 (74.6%)). Problems with usual activity increase across disease stages with a peak in stage III (*n* = 15 (79%)), and are reported by all patients in stage IV and V. Approximately half of all patients (*n* = 41 (51.2%)) experience *pain/discomfort*, but only three patients, one in each disease stage III, IV and V, report major pain/discomfort. A total of 56 (68.2%) patients report slight or major problems with *depression/anxiety*. In stage I most patients report no problems (*n* = 9 (75%)), while half or more report slight problems in all other stages (Stage II: *n* = 16 (73%), Stage III: *n* = 10 (53%), Stage IV (*n* = 7 (47%) and Stage V (*n* = 11 (65%)).

Overall self-reported HRQoL measured by EQ-5D VAS (*N* = 82), shows an average score of 52.1 (SD = 26.1), and decreases across disease stages with mean and SD values of 83 and 16.4 for stage I, 57.9 and 20.3 for stage II and 49.3 and 23.5 for stage III, and stable scores for Stage IV and V (*N* = 15) (mean (SD) values 35 (25.5) & 38.3 (20.9) respectively).

### The relationship between unmet needs and HRQoL

**Fig. 2:**
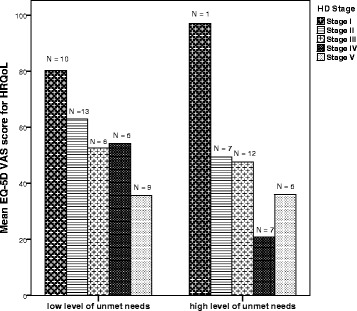
Bar-graph of average HRQoL scores across disease-stage for low vs. high levels of unmet needs

Figure 2 shows the decline in HRQoL measured by EQ-5D VAS score for the five disease stages for patients with unmet needs for healthcare and social support services (*N* = 73), divided into patient with a low level (median score for total unmet needs 1 – 6) and a high level (median score for total unmet needs > 6) of unmet needs. Overall, patients with a high level of unmet needs have lower HRQoL.

Results of the hierarchical regression analysis investigating the association between level of unmet needs for healthcare and social support services and HRQoL are presented in Table 3. The collinearity diagnostics suggested an acceptable degree of collinearity (VIF 1.1 – 2.6, and < 5). Cooks’ distance (D max = 0.205) indicated that no single case in the data induced undue influence on the model. The level of unmet needs was entered at the first step with statistically significant effect, explaining 9% of the total variance in HRQL. When the clinical disease characteristics (disease duration, TFC score, comorbidity and informant variable) were entered into the second step, the amount of explained variance improved with 30%. Only TFC showed significant effect. The model was controlled for demographics (age and education) which explained only 2% of variance. The β value for the level of unmet needs was negative (− 0.228; *p* = 0.018) indicating that a higher level of unmet needs was associated with lower HRQoL whereas the β value of TFC score (0.564; *p* < 0.001), was positive indicating that a better functional ability corresponded to higher HRQoL. The model was validated by removing the eight patients for whom only a rating performed by informant was available from the analysis, revealing no significant changes to the reported regression model, with level of unmet needs remaining significantly associated with HRQoL and no significant change in explained variance of the model.

**Table 3:** Hierarchical multiple regressions of the total level of unmet needs on self-reported HRQoL (*N* = 81)

Step variables	Variables	*R* ^*2*^ Change	β	β (95% CI)
1. Level of unmet needs^a^		0.092 (*p* = 0.006)	−0.228 (*p* = 0.018)	−9.711–0.933
2. Clinical characteristics	Disease duration	0.302 (*p* < 0.001)	−0.196 (*p* = 0.105)	−12.634–1.222
	Informant		0.116 (*p* = 0.352)	−7.320–20.3
	Comorbidity		−0.152 (*p* = 0.114)	−17.99–1.959
	TFC		0.564 (*p* < 0.001)	1.677–5.133
3. Socio-demographic characteristics	Age	0.026 (*p* = 0.205)	0.116 (*p* = 0.253)	−0.194–0.725
	Education		−0.138 (*p* = 0.144)	−2.450–0.363

*R* ^*2*^ = 0.42; Adjusted *R* ^*2*^ = 0.364; ^a^1 is missing NPCS total score

## Discussion

This is the first study describing self-reported health status (HRQoL) of HD patients in *all* five disease stages. The lowest rated health status was found in moderate to advanced phases of HD (stages III-V), while patients in stage III showed the most wide-ranging health profile. The study also investigated the association between level of unmet needs for healthcare and social support services and HRQoL. The main results support the view that a higher level of unmet needs is related to lower HRQoL. The findings are discussed in detail below.

The study results highlight the significant burden of HD on HRQoL. These findings are consistent with previous studies on HD [11, 17, 21, 26, 31]. Patients in advanced disease stages reported the most extensive reduction in HRQoL. However, already in the middle phase (stage III) patients rated their HRQoL below the average of the present sample and of the sample in Hocaoglu et al. [26]. Furthermore, the most wide-ranged health profile was found for these patients. Patients in this phase represent a largely heterogenic group, due to higher variation in symptom presentation and disease progression [20]. The phase can be considered a transitional phase, where patients transition from being relatively independent to becoming increasingly dependent in various areas of daily life; thus resulting in these patients experiencing considerable difficulties. However, some patients may report experiencing no problems at all, while other experience major problems with mobility, usual activities, self-care, pain and discomfort and anxiety and depression.

HRQoL is a multidimensional phenomenon influenced by several factors. The HRQoL regression model in this study covered data on the level of unmet health service needs, disease duration, comorbidity and functional ability, informant in the study, patient age and education, explaining 42% of the variance in HRQoL. A possible explanation why a large proportion of the variance remained unexplained is that we did not evaluate in depth the clinical status of patients, such as presence of cognitive impairment, psychiatric symptoms and motor impairment.

Some of the remaining explanatory factors may also be related to environmental conditions such as local health care organization, costs and quality of delivered care and socio-economic inequities among patients. In general, the Norwegian universal public health care system is based on a principle of equal access for all citizens according to needs, and not according to wealth. However, a recently published Norwegian study on self-reported health care utilization in the general population found social inequalities in utilization of specialized health care services [32].

Evidence on health care utilization, patients’ needs for health care services and HRQoL in the HD population is sparse [20]. In a qualitative study of healthcare experiences of families affected by HD from Canada, complex needs for healthcare services and emotional support were found. Participants expressed frustration at the lack of knowledge about HD displayed by their family physicians [33]. Several suggestions to improve the quality of care to their families, including better education of healthcare professionals regarding the complex nature of HD and the provision of regular follow-up support, were offered.

The strong association between functional capacity and HRQoL found in this study is consistent with previous studies on HD and HRQoL [11, 18]. Furthermore, this study found that higher level of unmet needs for healthcare and social services were related to lower self-rated health status (HRQoL). Information about such needs in the general Norwegian population or in individuals with disabilities does not exist. However, it is worth mentioning that a study from Canada, where there is a health care system with a similar accessibility to the Norwegian system, found that adults (aged 20–64 years) with physical, sensory and cognitive disabilities reported more than three times as many unmet health care needs as their non-disabled counterparts [34].

The study results are also supported by studies on unmet healthcare and social services needs and HRQoL in other patient groups [35–40]. Investigating unmet health needs in patients with coronary heart disease results showed associations between physical and social needs and HRQoL [35]. Research within the field of cancer, revealed that in adolescents and young adults with cancer unmet support service needs were associated with lower overall HRQoL [36]. Also, cancer survivors with unmet supportive care needs in the physical, psychological and patient care domains were shown to have poorer HRQoL [37]. Trying to identify factors related to quality of life in people with severe mental illness, researchers found the strongest predictors to be unmet basic, social and functioning needs [38]. A study on patients with dementia in residential care uncovered that sensory and physical disability needs, mental health and social needs were often unmet [39]. Taken together, the studies highlight that identifying healthcare needs is a vital part of providing comprehensive healthcare due to impact on HRQoL.

This study has limitations that should be addressed. A cross-sectional study design prevents us from discussing any causal relationship between independent variables and HRQoL. Future studies with a larger sample and longitudinal design need to be carried out in order to further tease apart the associations and predictors of HRQoL including disease specific aspects and level of unmet healthcare needs. Several studies on HD have used the SF-36 when investigating associations between disease specific symptoms and HRQoL. The SF-36 has demonstrated good validity in the context of HD [13]. Our study specifically focused on including HD patients in moderate to advanced disease stages, often underrepresented in HD research. The EQ-5D VAS score for HRQoL includes less complicated questions and applying a VAS Scale can be considered easier to administrate to advanced patients, compared to SF-36. While being a self-reported measure of HRQoL, eight EQ-5D questionnaires for advanced HD patients were completed on behalf of the patient by the primary family caregiver or professional carer, as the patients were not able to fill out the questionnaires themselves. Hocaoglu et al. [26] found good correlations between proxy and patient ratings of HRQoL in HD, but was lower for middle stages of HD compared to early and advanced stages on their HD-HRQoL measure. If a patient needed assistance from their primary carer to fill out the form, the carer was specifically instructed to *assist* in order to ensure best reflection of the patient’s rating. Further, our model did not change when we analyzed the data without the eight patients for whom only a rating by informant was obtained. We used a generic measure, which is less complex and well correlated with HD-HRQoL measures in a validation study [29]. Although we acknowledge our study’s limitations, we included a *relatively large group of patients in the middle to advanced stages* of HD: thus comprising a *relatively representative* sample of the HD population, covering the whole spectrum of disease.

## Conclusions

The present findings of the association between level of unmet needs for healthcare and social support services and overall HRQoL might have potential benefits for clinical practice where comprehensive care is targeted. In order to improve functioning and HRQoL of patients with HD, it is important that clinicians assess, record and monitor healthcare and social support service needs, as well as follow up that needs are met. Building partnerships with family caregivers may improve exchange of information and facilitate tailored health care delivery [41]. Our findings underline the importance of continuity of care through the whole disease spectrum (early to advanced HD) acknowledging the complex and changing nature of this disease [8, 9, 42].

## Abbreviations

CI: Confidence Interval
FAS: Functional Assessment Scale
HD: Huntington’s disease
HRQoL: Health-related Quality of Life
IQR: Interquartile Range
IS: Independence Scale
NPCS: Needs and Provision Complexity Scale
SD: Standard deviation
TFC: Total Functional Capacity
UHDRS: Unified Huntington’s Disease Rating Scale
VAS: Visual Analogue Scale
